# PD169 Is China Embracing Innovation? An Analysis Of Early Access In The Boao Lecheng International Medical Pilot Zone

**DOI:** 10.1017/S0266462324003957

**Published:** 2025-01-07

**Authors:** Katherine Leong, Richard Macaulay

## Abstract

**Introduction:**

Since 2018, medicines approved by regulators outside of China (e.g., the United States Food and Drug Administration) can be used in China’s Boao Lecheng International Medical Pilot Zone if approved by the Hainan authority. In 2019, the Implementation Plan for the Clinical Real-World Data Application Pilot was also introduced to facilitate real-world data generation in Boao Lecheng. This research examined the status of authorized medicines in Boao Lecheng.

**Methods:**

Information on authorized medicines and insurance coverage was extracted from the Boao Lecheng Administration Bureau’s website and official WeChat account. Regulatory information was extracted from the National Medical Products Administration (NMPA) website on 31 October 2023.

**Results:**

To date, 101 medicines have been authorized for use in Boao Lecheng. Of these, 45 (45%) were included in the Lecheng Global Special Drug Insurance Scheme, which provides commercial supplementary insurance for Hainan citizens, and 28 (28%) had received NMPA approval. In addition, 10 (10%) were included in Boao Lecheng’s Clinical Real-World Data Application Pilot, four of which have since received NMPA marketing authorizations using the real-world data generated to support their applications (pralsetinib, trilaciclib, inclisiran, and the fluocinolone acetonide intravitreal implant). Three of these four medicines passed preliminary review for the National Reimbursement Drug List (NRDL) in 2022, and one was listed on the NRDL in 2023.

**Conclusions:**

Boao Lecheng has become a channel for providing foreign medicines with early access to the Chinese market, with a considerable proportion being covered by commercial supplementary insurance or subsequently obtaining NMPA approval. Although few products have gone through Boao Lecheng’s real-world data pilot to date, early examples show that it offers a route for local real-world data collection to support NMPA marketing authorizations.

